# A novel pyroptosis-related prognostic lncRNAs signature, tumor immune microenvironment and the associated regulation axes in bladder cancer

**DOI:** 10.3389/fgene.2022.936305

**Published:** 2022-08-08

**Authors:** Xiaocong Mo, Di Hu, Yin Li, Aitao Nai, Feng Ma, Shoaib Bashir, Guoxia Jia, Meng Xu

**Affiliations:** ^1^ Department of Oncology, The First Affiliated Hospital of Jinan University, Jinan University, Guangzhou, China; ^2^ Department of Neurology and Stroke Centre, The Fist Affiliated Hospital of Jinan University, Guangzhou, China

**Keywords:** bladder cancer, pyroptosis, lncRNA, immune environment, prognosis, risk model, ceRNA

## Abstract

Bladder cancer (BC) is the most common malignancy of the urinary system. Pyroptosis is a host programmed cell death. However, the effects of pyroptosis-related lncRNAs (PRLs) on BC have not yet been completely elucidated. In this study, a prognostic PRLs model and two ceRNA networks were established using sufficient bioinformatics analysis and preliminary RT-qPCR validation *in vitro*. 6 PRLs were identified to construct a prognostic model. Then, the prognostic model risk score was verified to be an effective independent factor (Training cohort: Univariate analysis: HR = 1.786, 95% Cl = 1.416-2.252, *p* < 0.001; multivariate analysis: HR = 1.664, 95% Cl = 1.308-2.116, *p* < 0.001; testing cohort: Univariate analysis: HR = 1.268, 95% Cl = 1.144-1.405, *p* < 0.001; multivariate analysis: HR = 1.141, 95% Cl = 1.018-1.280, *p* = 0.024). Moreover, ROC and nomogram were performed to assess the accuracy of this signature (1-year-AUC = 0.764, 3-years-AUC = 0.769, 5-years-AUC = 0.738). Consequently, we evaluated the survival curves of these 6 lncRNAs using Kaplan–Meier survival analysis, demonstrating that MAFG-DT was risk lncRNA, while OCIAD1-AS1, SLC25A25-AS1, SNHG18, PSMB8-AS1 and TRM31-AS1 were protective lncRNAs. We found a strong correlation between PRLs and tumor immune microenvironment by Pearson’s correlation analysis. As for sensitivity of anti-tumor drugs, the high-risk group was more sensitive to Sorafenib, Bicalutamide and Cisplatin, while the low-risk group was more sensitive to AKT.inhibitor.VIII, Salubrinal and Lenalidomide, etc. Meanwhile, we identified lncRNA OCIAD1-AS1/miR-141-3p/GPM6B and lncRNA OCIAD1-AS1/miR-200a-3p/AKAP11 regulatory axes, which may play a potential role in the progression of BC.

## Introduction

Bladder cancer (BC) is the most common malignancy of the urinary system with the highest incidence (Lenis et al., 2020), which originates from transitional cells of bladder urothelium (Martinez Rodriguez et al., 2017). Though many biomarkers have been discovered to predict the prognosis of BC (Tan et al., 2018; Zhang et al., 2018), they still exist many deficiencies (Shi and Tian, 2019).

Pyroptosis is a host cell death pathway stimulated by a series of microbial infections and non-infectious stimuli (Bergsbaken et al., 2009). It is relevant to inflammasome-related diseases and compounds (Frank and Vince, 2019; Tsuchiya, 2020). Pyroptosis differs from other cell death forms in morphology and mechanism; caspase-1 dependence is a defining characteristic (Bergsbaken et al., 2009). In recent years, a large number of studies have found a close relationship between pyroptosis and human cancer. For example, PD-L1 promotes cancer cell pyroptosis by mediating the expression of gasdermin C (Hou et al., 2020). Polyphyllin VI induces caspase-1-mediated pyroptosis in lung cancer (Teng et al., 2020). *α*-NETA induces pyroptosis by target regulating GSDMD/caspase-4 signal way in epithelial ovarian cancer (Qiao et al., 2019). However, it is currently unclear in bladder cancer.

LncRNA is a new-found functional ncRNA. Plenty of studies have confirmed that lncRNAs play roles in the development of BC. For example, lncRNA CASC11 promotes the proliferation of bladder cancer cells by sponging miRNA-150 (Luo et al., 2019), and exosomal lncRNA LNMAT2 promotes lymphatic metastasis in bladder cancer (Chen C. et al., 2020). Besides, lncRNA CCAT1 accelerates bladder cancer cell migration, proliferation, and invasion (Zhang et al., 2019). Recently, the regulation of lncRNAs on pyroptosis has been extensively studied. A study showed that the interference of lncRNA XIST inhibits lung cancer progression by stimulating the pyroptosis (Liu et al., 2019). Additionally, lncRNA SNHG7 inhibits NLRP3-dependent pyroptosis by regulating the miR-34a/SIRT1 signal pathway in liver cancer (Chen Z. et al., 2020). However, the involvement of pyroptosis-correlated lncRNAs in BC is unclear.

## Materials and methods

### Data collection

Transcriptional data and mutation data for BC were obtained from TCGA (https://portal.gdc.cancer.gov/), copy number variation (CNV) data from the UCSC Xena website (http://xena.ucsc.edu/) and clinical characteristics of BC patients from GEO (https://www.ncbi.nlm.nih.gov/geo/).

### Identification of pyroptosis-correlated genes

52 pyroptosis-correlated genes were identified based on previous reports(Xu et al., 2021; Dong et al., 2021; Li et al., 2021; Shen et al., 2021). 29 mRNAs were selected (*p < 0.05, **p < 0.01 and ***p < 0.001) by R software. A PPI network of the 29 differentially expressed genes (DEGs) was established by STRING (https://string-db.org/) with the interaction score set as 0.9. In addition, the network was visualized through Cytoscape software 3.7.2.

### KEGG and GO enrichment analysis

The GO (http://www.geneontology.org/) and the KEGG (http://www.genome.jp/kegg/) enrichment analyses were conducted. The GO database and KEGG were used to identify the biological characteristics and signaling pathway of pyroptosis-correlated genes.

### Construction and clinical meaning of the model

LncRNAs of these differentially expressed genes were identified (|R|>0.3 and *p* < 0.001). The included cases (*n* = 406) were classified into training and validation cohorts. Pyroptosis-correlated lncRNAs were selected using LASSO Cox algorithm. Using the regression coefficient (*β*), the risk score = *β*_1_*Expression_1_+*β*_2_*Expression_2_ + … + *β*_n_*Expression_n_. Moreover, survival curves of these lncRNAs were plotted using Kaplan–Meier survival analysis. *p* < 0.05 was considered statistically significant. Univariate and multivariate Cox regressions were used to identify the clinical meaning of the prognostic model. A nomogram was constructed using clinical factors and patient’s risk score, and shows risk scores of 1, 3, and 5 years survival rates.

### The construction of PCA and GSEA analysis

PCA was performed to converge BC patients according to the expression patterns of pyroptosis-correlated genes. In addition, the distribution of patients was visualized by 3D scatter plots. GSEA analyzed the differences of biological pathways.

### Analyses of immune cells and immune-related pathways

Single-sample gene set enrichment analysis (ssGSEA) was performed to assess the infiltration fractions of immune cells and the activities of immune-related pathways using the “gsva” R package. Moreover, ssGSEA was performed to assess the correlation between the risk model and immune cells infiltration, as well as PRLs and immunity.

### Analyses of potential drug candidates for BC

Potential drug candidates were selected using the “pRRophetic” package. We obtained some drugs that may become candidates for the treatment of BC according to the expression matrix of patients.

### Construction of competing endogenous RNA network

To identify the molecular mechanism of pyroptosis-correlated lncRNAs in BC, we established a ceRNA network. Mircode (www.mircode.org) and LncRNABase database (http://starbase.sysu.edu.cn/mirLncRNA.php/) were used to predict the miRNA. And we explored its downstream mRNA targets to construct the miRNA-mRNA axis. TargetScan (http://www.targetscan.org/vert_72/), Mircode (http://www.mircode.org/), and miRDB databases (http://mirdb.org/) were utilized to predict mRNA targets interacting with miRNAs. Moreover, we calculated the expression and prognostic value of these miRNA and mRNA targets. *p* < 0.05 was considered statistically significant.

### RT-qPCR analysis

Total RNA was isolated from cultured cells using TRIzol (Invitrogen). cDNA was obtained by reverse transcription using SuperScript III First-Strand cDNA System (Invitrogen, Thermo Fisher Scientific, Inc., USA). The GPM6B sense primer was 5′-TGA​GCG​AGG​TGA​TAC​AAC​TGA​TGC-3′, and the antisense primer was 5′-GCC​ACT​CCA​AGC​ACA​TAG​GTG​AG-3'. The AKAP11 sense primer was 5′-CAC​GTT​ACA​CCA​GAA​TTG​CCT​A-3′, and the antisense primer was 5′-TGG​TCT​CAG​ACA​CTC​GGA​AC-3'. RT-qPCR was performed using a 7900HT fast Real-time PCR system (Life Technologies, Carlsbad, CA). The RNA expression data were calculated using the comparative threshold cycle (2^−ΔΔCq^) method.

### Statistical analysis

One-way analysis of variance and the *t*-test were used. SPSS 26.0 software and GraphPad Prism 9.3.0 were used to analyze data. All data are presented as mean ± SD. All experiments were repeated three times. *p* < 0.05 was statistically significant.

## Results

### The expression of pyroptosis-related genes in BC

The expression levels of 52 genes with regard to pyroptosis were compared between BC and tissues, and 29 pyroptosis-related genes were identified as DEGs*.* Compared with normal tissues, 23 genes (AIM2, BAK1, BAK, CASP3, CHMP7, CASP5, CASP6, IL-6, CASP8, CHMP2A, CHMP4A, CHM4B, CHMP4C, CYCS, GPX4, GSDMD, TP63, HMGB1, IL1A, TP53, PYCARD, PLCG1, GSDMB, NLRP2, NLRP7) were detected to be up-regulated, while 6 genes (CHMP3, ELANE, NLRP3, NLRP1) were down-regulated in BC group (Figure 2A). PPI presented the interaction with the interaction score set as 0.9 (Figure 2B), and the hub genes are shown in Figure 2C. In addition, Figure 2D shows the relationship network between all pyroptosis-related genes in another way, and both confirmed the highly complex specific interaction patterns among these pyroptosis-related genes (Figures 2C,D). Interestingly, it can be seen from the waterfall chart of mutations in all 29 pyroptosis-correlated genes that TP53 had the highest mutation frequency (Figure 2E). Most of the 29 DEGs show a trend that the frequency of copy number “gain” was greater than the frequency of “loss”, especially in AIM2, TP63, and GSDMB (Figures 2F,G).

**FIGURE 1:**
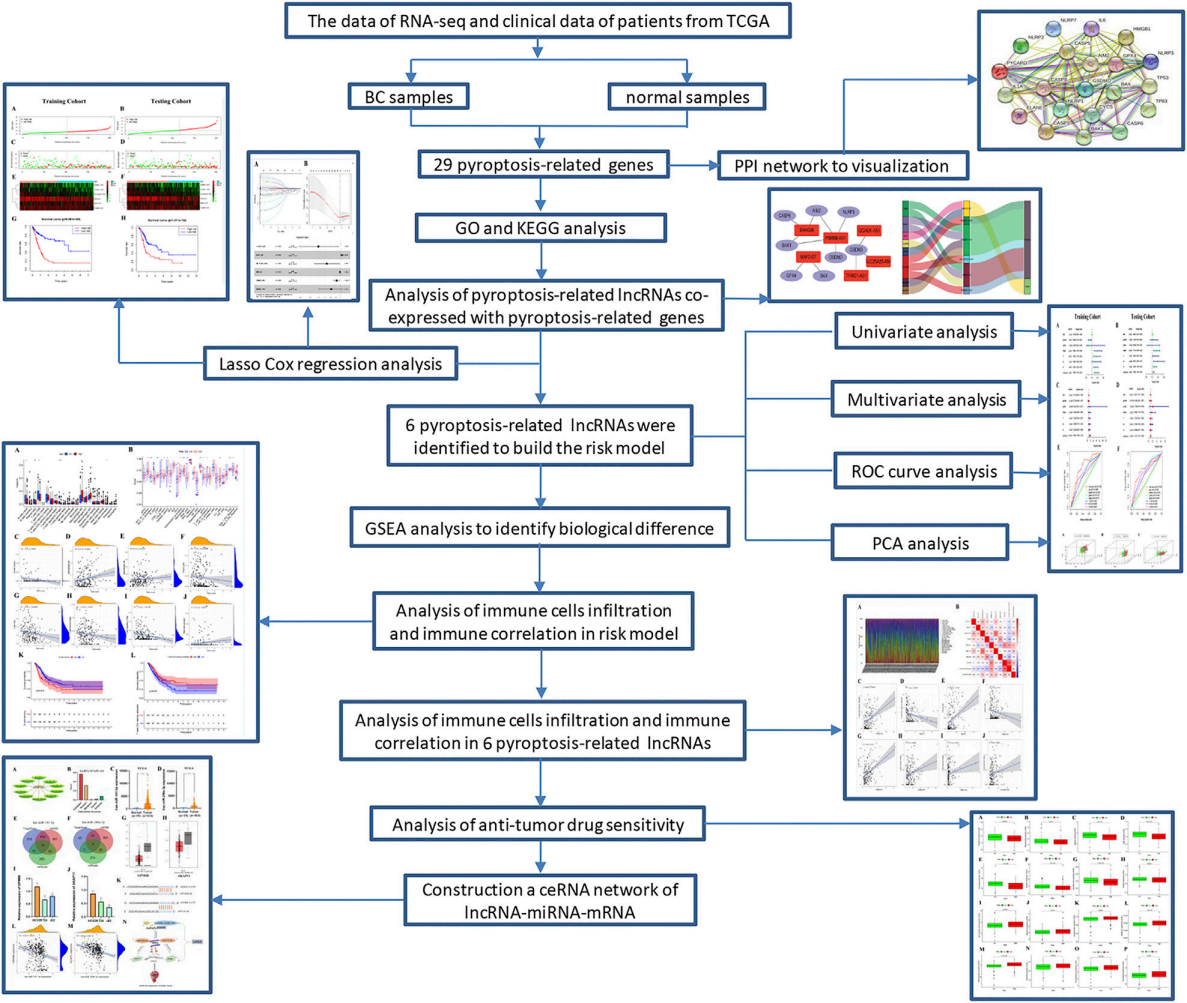
Schematic diagram of the study.

**FIGURE 2:**
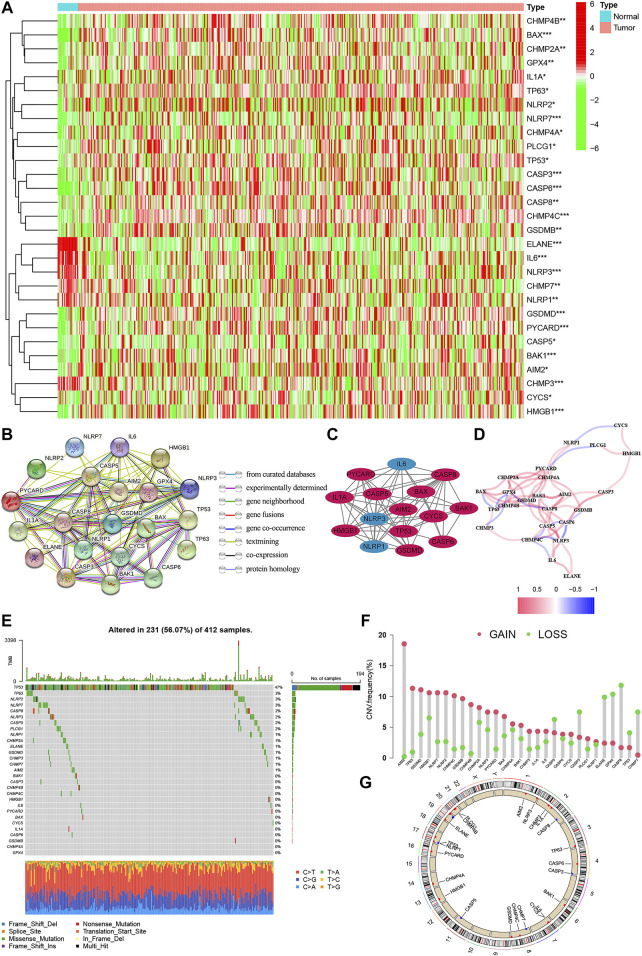
The expression of pyroptosis-related genes in BC. **(A)** Heatmap of the 29 pyroptosis-correlated genes in BC and normal tissues. 23 genes were up-regulated, while 6 were down-regulated in the BC group. **(B)** PPI network of the interaction with the interaction score sett as 0.9. **(C)** The hub genes were obtained from the PPI network with 15 genes using the MCODE plug-in. **(D)** The relationship network of the pyroptosis-correlated genes. Red and Blue lines: positive and negative correlation. **(E,F)**: Genetic alterations and CNV variation frequency of pyroptosis-correlated genes in BC, and TP53 had the highest mutation frequency. **(G)** Location of CNV alterations in BC. CNV, copy number variation; PPI, protein-protein interaction network.

### Biological functional research of pyroptosis-related genes

**FIGURE 3:**
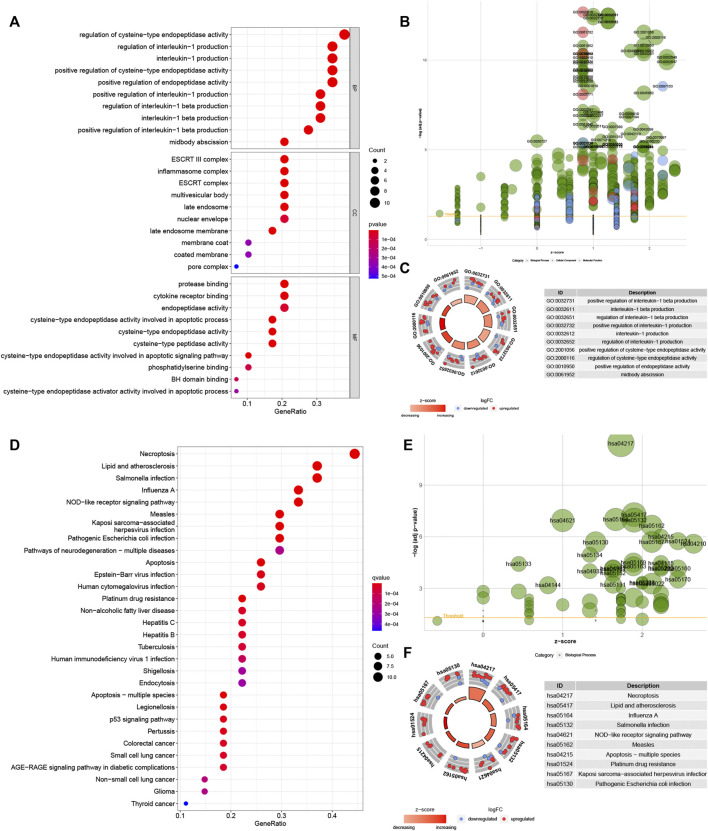
Biological functional enrichment research of pyroptosis-related genes. Functional enrichment analysis of PRGs. **(A–C)**: GO enrichment of PRGs in biological processes (BP), cellular component (CC) and molecular function (MF). **(D–F)**: The enriched KEGG pathways of PRGs. PRG, pyroptosis-related gene; GO, gene ontology; KEGG, Kyoto Encyclopedia of Genes and Genomes.

GO analysis showed these genes were enriched in “regulation of cysteine-type endopeptidase activity”, “regulation of interleukin-1 production”, “interleukin-1 production” and “midbody abscission” in biological processes (BP). These genes were enriched in “ESCRT III complex”, “inflammasome complex”, “multivesicular body” and “late endosome” in the cellular component (CC). These genes in molecular function (MF) were enriched in “protease binding”, “cytokine receptor binding”, ‘peptidase regulator activity” and “endopeptidase activity cysteine-type involved in apoptotic process” (Figures 3A–C). Meanwhile, KEGG analysis showed these genes were involved in “necroptosis”, “lipid and atherosclerosis”, “salmonella infection” and ‘influenza A’ (Figures 3D–F).

### Co-expression network construction

**FIGURE 4:**
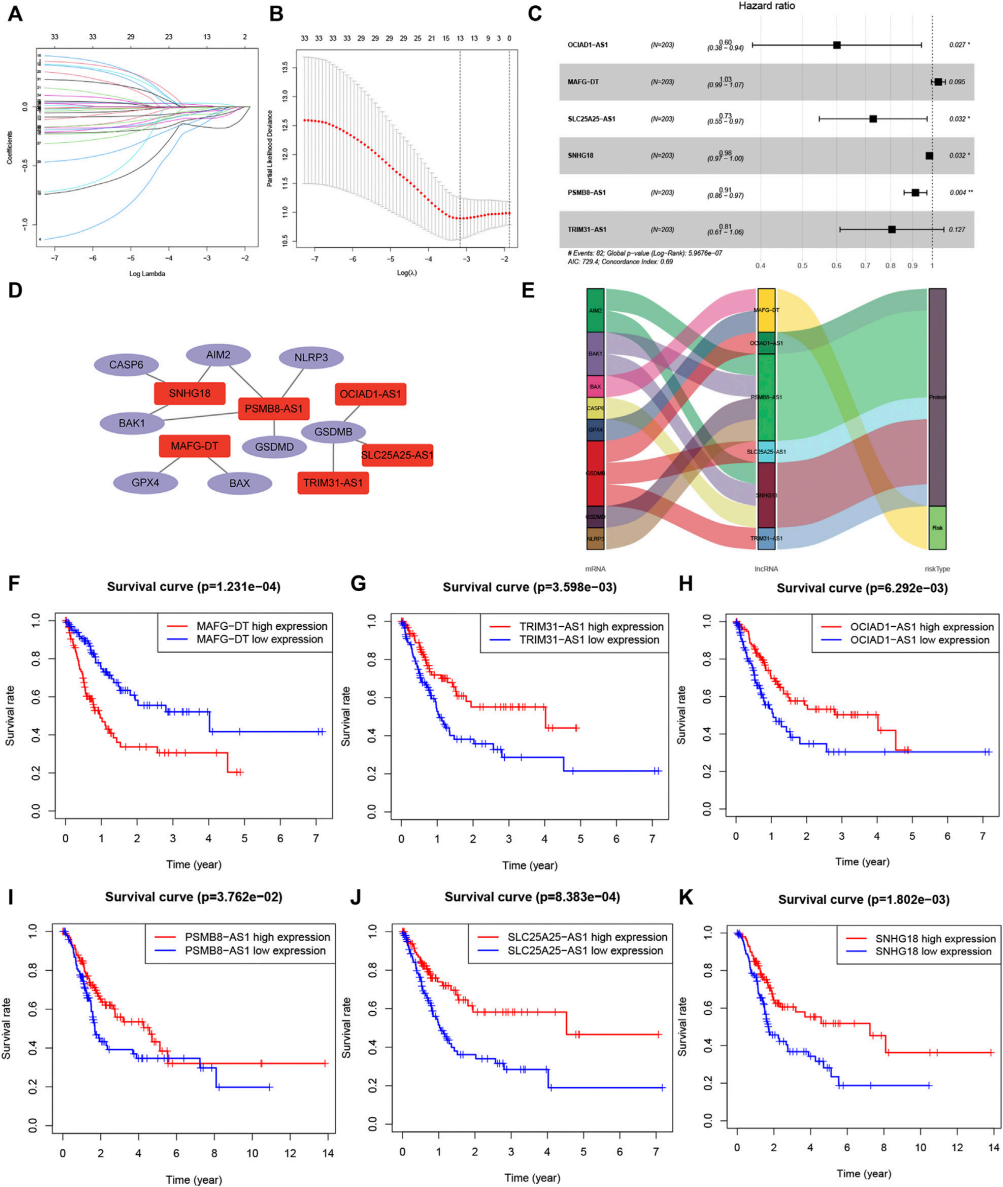
Co-expression network construction. **(A,B)**: LASSO Cox algorithm to establish a prognosis model; 13 PRLs were good candidates for constructing the prognostic model; **(C)**: 6 lncRNAs were selected. **(D)**: The co-expression structure between PRLs and genes. **(E)**: Sankey diagram of the co-expression network. **(F–K)**: Kaplan–Meier survival analyzed the overall survival of 6 lncRNAs, OCIAD1-AS1, MAFG-DT, SLC25A25-AS1, SNHG18, PSMB8-AS1 and TRIM31-AS1. PRL, pyroptosis-related lncRNA.

The included cases (*n* = 406) were classified into training (*n* = 203) and validation (*n* = 203) cohorts. LncRNAs related to 29 pyroptosis-related genes were screened out using Pearson correlation method. Then, we used the univariate Cox regression analysis and LASSO Cox algorithm and found 13 PRLs (Figures 4A,B). Total 6 lncRNAs, including OCIAD1-AS1, MAFG-DT, SLC25A25-AS1, SNHG18, PSMB8-AS1 and TRIM31-AS1, were selected, and the global *p*-value = 5.9676e-7 (Figure 4C). The co-expression network was presented in Figure 4D. Among these 6 PRLs, MAFG-DT was identified as risk lncRNA, while OCIAD1-AS1, SLC25A25-AS1, SNHG18, PSMB8-AS1 and TRIM31-AS1 were identified as 5 protective lncRNAs (Figure 4E). In addition, Figures 4F–K indicated that high-expression level of 5 lncRNAs (OCIAD1-AS1, SLC25A25-AS1, SNHG18, PSMB8-AS1 and TRIM31-AS1) tended to better overall survival compared to the low-expression groups. However, the contrary result was observed in lncRNA MAFG-DT.

### Construction of the risk model in BC patients

**FIGURE 5:**
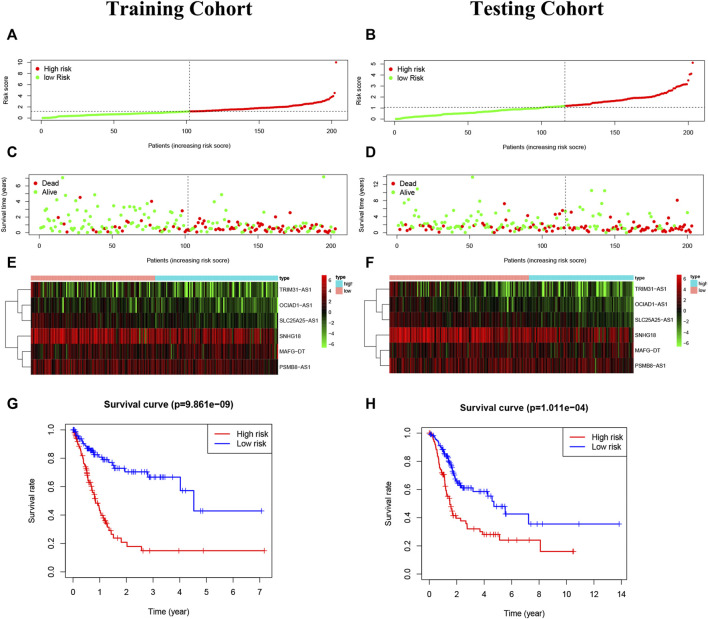
Construction of the risk model. **(A,B)**: The risk score in the training cohort and validation cohort. **(C,D)**: The survival status of BC patients. Green: survival, the red: death. The higher the risk score, the more intensive of death status. **(E,F)**: The heatmap of 6 pyroptosis-related lncRNAs. **(G,H)**: The Kaplan-Meier analysis in the two risk groups, and high-risk patients had worse overall survival than low-risk patients.

The risk score = OCIAD1-AS1×(-0.509,246)+MAFG-DT×0.031927 + SLC25A25-AS1×(-0.315,655)+SNHG18×(-0.015526)+PSMB8-AS1×0.090197 + TRIM31-AS1×0.215,654. BC patients were divided into two groups on the basis of median risk score (Figures 5A–D). The expression degrees of 6 lncRNAs were presented by heatmap (Figures 5E,F). Moreover, patients of high-risk had worse overall survival by Kaplan-Meier (*p* < 0.05) (Figures 5G,H).

### Prognosis value of model lncRNAs in BC

**FIGURE 6:**
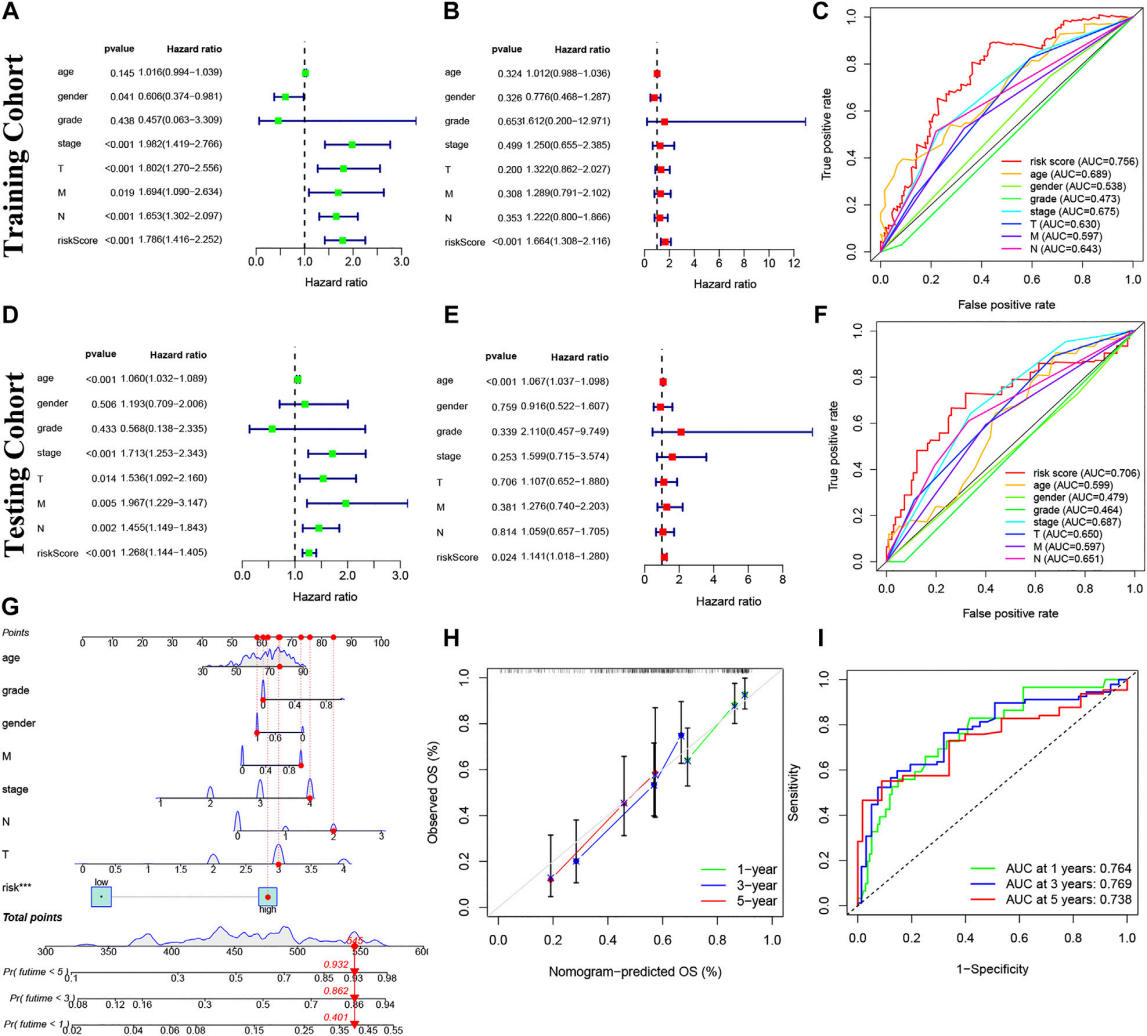
Prognosis value of model lncRNAs in BC. **(A,D)**: Univariate analysis. **(B,E)**: Multivariate analysis. Panel **(C,F)**: The ROC curves (Training cohort: AUC = 0.756; Testing cohort: AUC = 0.706). **(G)** Nomogram of 1-, 3- and 5-years survival. **(H)** Calibration curve. **(I)** The results of ROC curves in predicting of BC survival rates at 1-, 3- and 5- years (1-year-AUC = 0.764, 3-years-AUC = 0.769, 5-years-AUC = 0.738). ROC, receiver operator characteristic; AUC, area under curve.

Our risk model was an independent factor in BC (Training cohort: Univariate analysis: HR = 1.786, 95% Cl = 1.416-2.252, *p* < 0.001; multivariate analysis: HR = 1.664, 95% Cl = 1.308-2.116, *p* < 0.001; testing cohort: Univariate analysis: HR = 1.268, 95% Cl = 1.144-1.405, *p* < 0.001; multivariate analysis: HR = 1.141, 95% Cl = 1.018-1.280, *p =* 0.024) compared with other clinical factors (Figures 6A,B,D,E). Moreover, the risk score is more accurate than other clinical features (Training cohort: AUC = 0.756; Testing cohort: AUC = 0.706) (Figures 6C,F). For internal validation, the prediction nomogram shows that the overall survival rate can be predicted relatively well in comparison with the ideal model. The nomogram could be used to examine potential associations between clinical features and patient prognosis. (Figures 6G,H). Meanwhile, the risk model had a high sensitivity and efficacy (1-year-AUC = 0.764, 3-years-AUC = 0.769, 5-years-AUC = 0.738) (Figure 6I).

### PCA analyses and important pathways of the risk model

**FIGURE 7:**
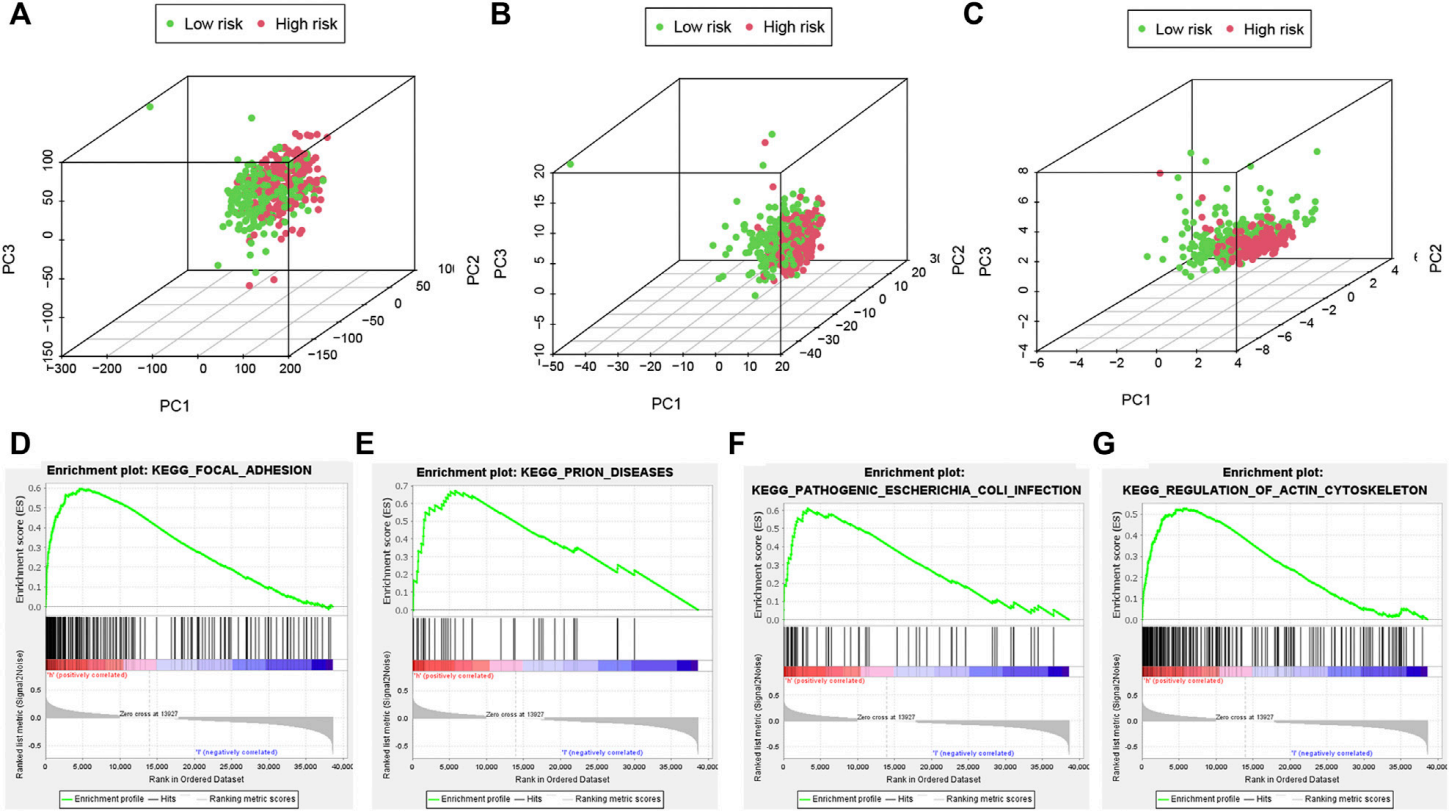
PCA analyses and important pathways of the risk model. The PCA visualized the different distribution patterns of patients in **(A,B)**: all pyroptosis-related genes, lncRNAs and **(C)** 6 pyroptosis-related lncRNAs using 3D scatterplot. PCA analyses show the risk model could clearly distinguish the two risk groups (high and low) of patients. **(D–G)**: GSEA analysis showed important pathways in gene expression of two risk groups. PCA, principal component analysis; GSEA, Gene Set Enrichment Analysis.

Different distribution patterns of patients in all pyroptosis-correlated genes, all pyroptosis-correlated lncRNAs and 6 pyroptosis-correlated lncRNAs were visualized by a 3D scatterplot of PCA, respectively (Figures 7A–C). These outcomes show that the risk model could clearly distinguish the two risk groups (high and low) of patients. Moreover, GSEA analysis was performed to search important pathways in gene expression of two risk groups between high and low. It was suggested that gene expression of the two risk groups was differentially related to immune-related pathways (Figures 7D–G).

### Analysis of immune activity in different groups

**FIGURE 8:**
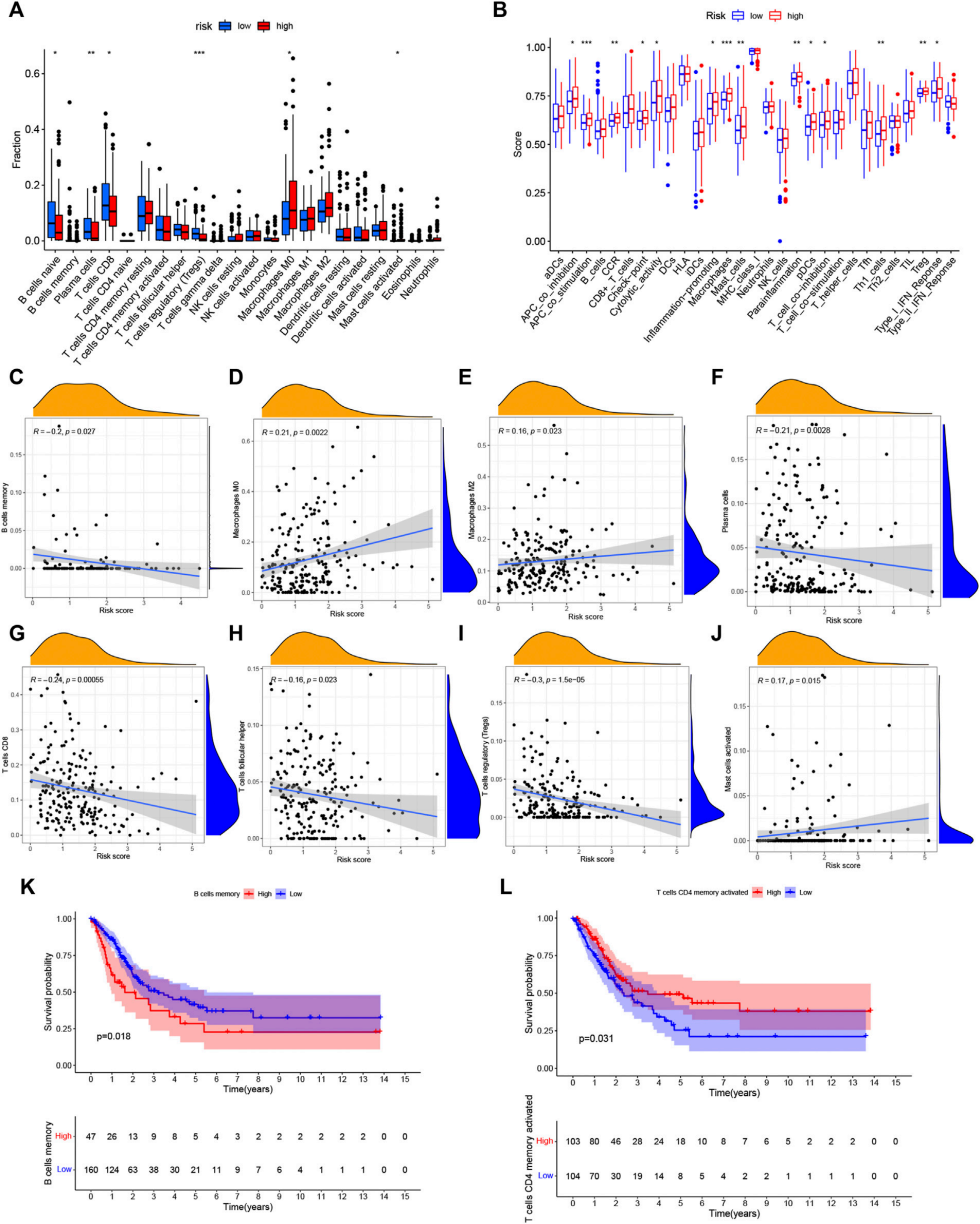
Analysis of immune activity. **(A)** Comparison of immune cells. And 6 of these immune cell types were differentially expressed in two risk groups (*p* < 0.05). **(B)** Comparison of immune-correlated pathways. The high-risk group of the TCGA cohort significantly had a higher score than the low-risk group. **(C–J)**: There was a positive correlation between the survival outcome of BC patients and the high degrees of Macrophages (M0, M2) and Mast cells, while there was a negative correlation of CD8 T cells, B cells, T cells follicular helper, Tregs and plasma cells **(K,L)**: Kaplan–Meier survival analyzed the overall survival of two groups in B cells memory and T cells CD4 memory activated. *p < 0.05, **p < 0.01; ***p < 0.001.

The ssGSEA algorithm was performed to compare 16 different immune cell types in BC, 6 of these immune cell types were differentially expressed (*p* < 0.05) (Figure 8A). Moreover, there were a different immune-score among these two groups (Figure 8B). Furthermore, the relationship of immune cells is showed in Figures 8C–J. There was a negative correlation between the survival outcome of BC patients and the high degrees of CD8 T cells, B cells, T cells follicular helper, Tregs, and plasma cells. Interestingly, we discovered that some immune cells (B cells memory and T cells CD4 memory activated) were significantly associated with the overall survival of BC patients (Figures 8K,L).

### Correlation analysis of 6 pyroptosis-related lncRNAs and immunity

**FIGURE 9:**
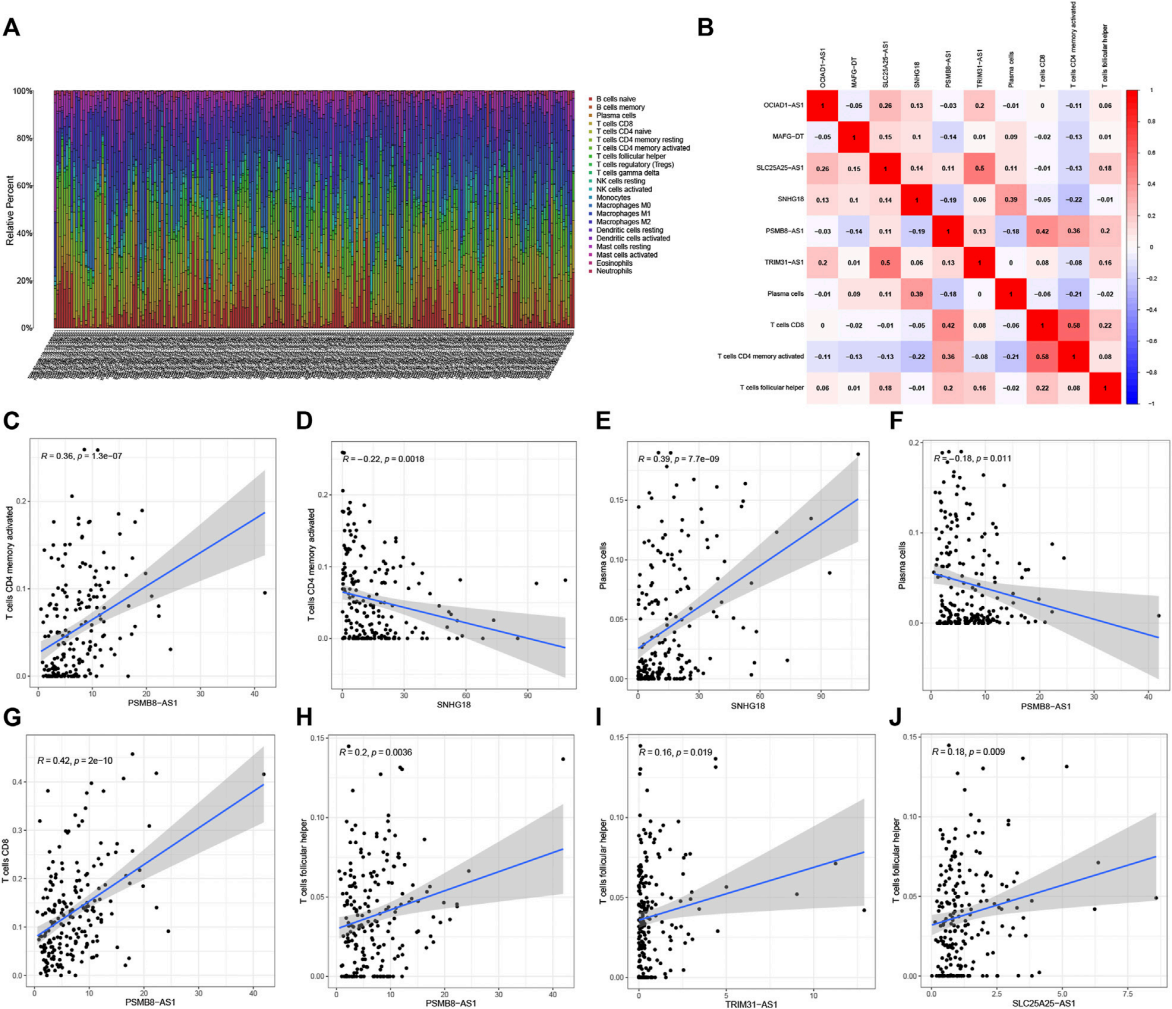
Correlation analysis of 6 PRLs and immunity. **(A)** The infiltration degrees of immune cells in BC samples. **(B)** Heatmap reflected the distribution of the 6 PRLs and immune cells. **(C,D)**: There was a positive correlation between T cells CD4 memory activated and lncRNA PSMB8-ASI, while there was a negative correlation between T cells CD4 memory activated and lncRNAs SNHG18. **(E,F)**: Plasma cells contained a positive correlation with lncRNA SNHG18 and negative correlation with lncRNA PSMB8-ASI. **(G)** T cells CD8 involved a positive correlation with lncRNA PSMB8-ASI. **(H–J)**: T cells follicular helper and 3 lncRNAs (PSMB8-ASI, TRIM31-AS1and SLC25A25-AS1) presented a positive correlation distribution.

Figure 9A shows the degrees of infiltration of various immune cells in different BC samples, suggesting that the distributions of immune cells were widespread. In addition, the relationship between 6 PRLs and infiltration of immune cells is described in Figures 9B–J, revealing that T cells follicular helper and 3 lncRNAs (PSMB8-ASI, TRIM31-AS1and SLC25A25-AS1) presented a positive correlation distribution. In addition, there was a positive correlation between T cells CD4 memory activated and lncRNA PSMB8-ASI, while there was a negative correlation between T cells CD4 memory activated and lncRNAs SNHG18. Besides, plasma cells contained a positive correlation with lncRNA SNHG18 and a negative correlation with lncRNA PSMB8-ASI. Furthermore, T cells CD8 is positively correlated with lncRNA PSMB8-ASI.

### Difference analysis of anti-tumor drug sensitivity between high and low risk groups

**FIGURE 10:**
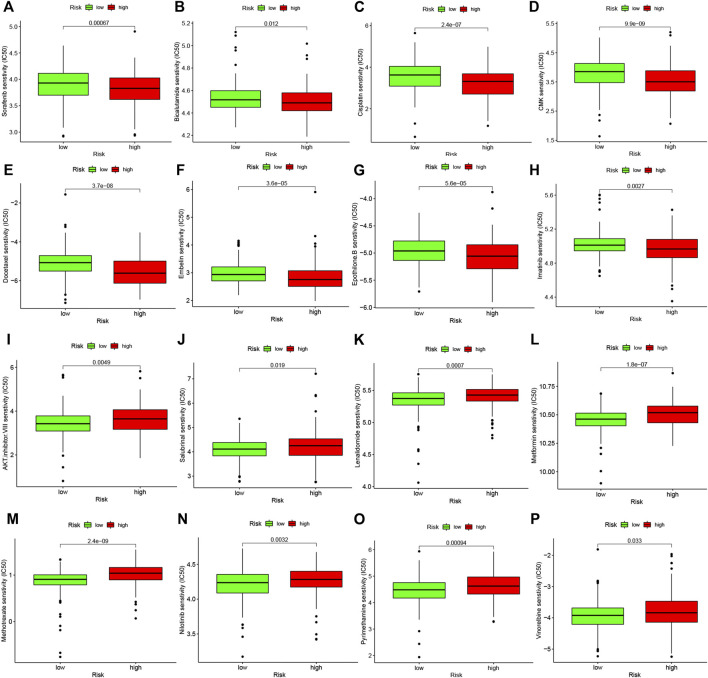
Difference analysis of anti-tumor drug sensitivity between high and low risk groups. **(A–H)**: The low-risk group was more sensitive to Sorafenib, Bicalutamide, Cisplatin, etc. **(I–P)**: The high-risk group was more sensitive to Camptothecin, Lenalidomide, Methotrexate, etc.

Studies on the sensitivity of anti-tumor drugs could enhance the development of future clinical treatment. The results indicated that the high-risk group was more sensitive to Sorafenib, Bicalutamide, Cisplatin, etc., as shown in Figure 10. Moreover, the low-risk group was more sensitive to AKT.inhibitor.VIII, Salubrinal, Lenalidomide, etc., as shown in Figure 10. These results are instructive for us to select specific drugs based on anti-tumor drug sensitivity.

### Construction of a regulatory axis of LncRNA-miRNA-mRNA

**FIGURE 11:**
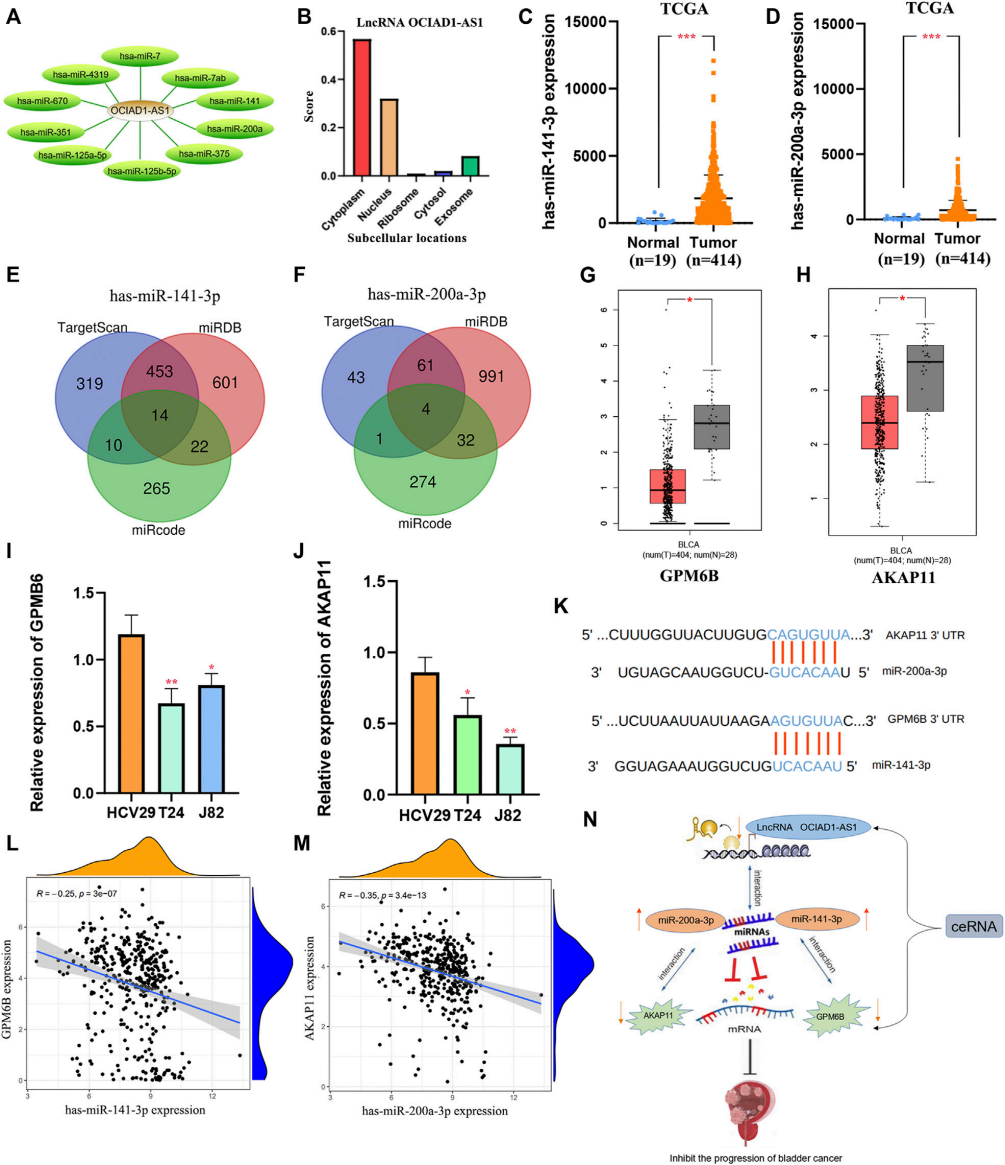
Construction of regulatory axes. **(A)** lncRNA OCIAD1-AS1 bound 10 miRNAs. **(B)** lncRNA OCIAD1-AS1 was concentrated in the cytoplasm than other subcells. **(C,D)**: Compared to normal tissues, miR-141-3p and miR-200a-3p were low-expressed in BC tissues. **(E,F)**: 14 mRNAs and 4 mRNAs were identified as downstream targets in miR-141-3p and miR-200a-3p, respectively, from miRDB, Mircode, and TargetScan database. **(G,H)**: GPM6B and AKAP11 were down-regulated in bladder cancer tissues compared to normal tissues by the GEPIA database. **(I,J)**: RT-qPCR results of GPM6B and AKAP11 in two human bladder cancer cell lines (J82 and T24) and a normal urinary tract cell line (HCV29). **(K)** The miR-141-3p and miR-200a-3p binding sites in BC. **(L,M)**: The correlation of GPM6B and AKAP11 with miR-141-3p and miR-200a-3p respectively in BC tissues. **(N)**: The ceRNAs of lncRNA OCIAD1-AS1/miR-141-3p/GPM6B and lncRNA OCIAD1-AS1/miR-200a-3p/AKAP11 regulatory axes. *p < 0.05, **p < 0.01; ***p < 0.001.

According to LncRNABase and Mircode databases, we found lncRNA OCIAD1-AS1 bound 10 miRNAs, which were miR-7, miR-7ab, miR-125a-5p, miR-141(miR-141-3p/5p), miR-200a (miR-200a-3p/5p), miR-375, miR-351, miR-125b-5p, miR-4319, and miR-670 (Figure 11A). MiR-141-3p and miR-200a-3p were reported to be highly expressed in bladder cancer (Ghorbanmehr et al., 2019, Tan S. et al., 2021), which was contrary to the expression of the target lncRNA OCIAD1-AS1 (Figures 11C,D). Surprisingly, we found that lncRNA OCIAD1-AS1 was concentrated in the cytoplasm compared with other subcells (Figure 11B). 14 mRNAs and 4 mRNAs were identified as downstream targets in miR-141-3p and miR-200a-3p respectively from miRDB, Mircode and TargetScan database (Figures 11E,F). We finally found that GPM6B and AKAP11 were down-regulated bladder cancer tissues compared to normal tissues using the GEPIA database (Figures 11G,H), which was consistent with the results of RT-qPCR (Figures 11I,J). In addition, the correlation analysis showed that GPM6B and AKAP11 were related to the expression of miR-141-3p and miR-200a-3p, respectively, in BC tissues. In other words, miR-141-3p and miR-200a-3p were highly-expressed, but GPM6B and AKAP11 were low-expressed in cancer tissues (Figures 11L,M). Thus, two ceRNA networks of lncRNA OCIAD1-AS1/miR-141-3p/GPM6B and lncRNA OCIAD1-AS1/miR-200a-3p/AKAP11 regulatory axes may play an indispensable role in the progression of BC (Figure 11N).

## Discussion

Our study systematically identified PRLs in BC. In the waterfall chart of mutations in these pyroptosis-correlated genes, TP53 had the highest mutation frequency. Mutations in TP53 elicit intratumoral T cell responses, which suggests that this protein is a candidate for anti-cancer immunotherapy (Chasov et al., 2021). In addition, the gene expression in these two risk groups was confirmed differentially related to immune-related pathways by GSEA analysis. Besides, we analyzed the difference in anti-tumor drug sensitivity between the two risk groups, founding that the high-risk group was more sensitive to Sorafenib, Bicalutamide, and Cisplatin. In contrast, the other group was more sensitive to AKT.inhibitor.VIII, Salubrinal, and Lenalidomide, etc.And we predicted and established two networks of lncRNA-miRNA-mRNA, which identified the signal axes of lncRNA OCIAD1-AS1/miR-141-3p/GPM6B and lncRNA OCIAD1-AS1/miR-200a-3p/AKAP11.

A number of models have been developed for prognostic prediction according to pyroptosis-related genes and clinical factors. For example, Xie et al. constructed a prognosis model of melanoma based on pyroptosis related genes. Cao et al. established a signature of pyroptosis-related genes of uveal melanoma, which can accurately guide the prognosis. On the other hand, numerous studies have confirmed the important role of lncRNAs in pyroptosis of different malignant cells. For example, Tan et al. reported that lncRNA HOTTIP could inhibit cell pyroptosis in ovarian cancer (Tan C. et al., 2021). Liu et al. discovered that lncRNA H19 mitigates oxidized low-density lipoprotein induced pyroptosis *via* caspase-1 in raw 264.7 cells (Liu et al., 2021). Liu et al. identified that lncRNA XIST could promote non-small cell lung cancer growth (Xu et al., 2020). Meanwhile, predictive models of pyroptosis-related lncRNAs have also been a hot research topic. For example, Liu et al. established a predictive model in uterine corpus endometrial carcinoma (Liu et al., 2022). And we established a predictive model using 6 pyroptosis-related lncRNAs, including SNHG18, SLC25A25-AS1, OCIAD1-AS1, MAFG-DT, TRIM31-AS1 and PSMB8-AS1. Meaningly, Li et al. found that the lncRNA SLC25A25-AS1 could significantly restrain proliferation and aggregation of colorectal cancer (CRC) cells, manifesting that lncRNA SLC25A25-AS1 played a biomarker role in prognosis (Li et al., 2016). Moreover, lncRNA SLC25A25-AS1 has also been reported in lung cancer (Chen et al., 2021) and prostate cancer (Wang Y. et al., 2021). In addition, lncRNA OCIAD1-AS1 was identified as a protective factor in BC patients with HR < 1 (Wang J. et al., 2021). LncRNA SNHG18 could facilitate the development of glioma cells (Zheng et al., 2019), which is the opposite of what we found in this project. Tong et al. revealed that the epithelial mesenchymal transition-related lncRNA PSMB8-AS1 was referred to as a prognostic marker and protective effector in bladder cancer (Wang Y. et al., 2021). In addition, we analyzed the difference in anti-tumor drug sensitivity between high-risk and low-risk groups, which would guide us to select specific drugs.

The lncRNA-correlated ceRNAs play a role in the development of cancers, but the lncRNA-related ceRNA is obscure in bladder cancer. Therefore, the novel network of lncRNA-miRNA-mRNA was constructed by using biological tools and the regulatory axes of lncRNA OCIAD1-AS1/miR-141-3p/GPM6B and lncRNA OCIAD1-AS1/miR-200a-3p/AKAP11 were predicted. In fact, some studies discovered that miR-141-3p and miR-200a-3p could promote bladder cancer progression (Liu et al., 2020). These reports supported our experimental results that the high expression of miR-141-3p and miR-200a-3p in BC. Our study predicted that lncRNA OCIAD1-AS1 might interact with miR-141-3p and miR-200a-3p. In addition, He et al. identified that GPM6B inhibited the malignant development of prostate cancer (Lin et al., 2020). We found that GPM6B and AKAP11 showed low expression levels in bladder cancer tissue compared to the normal group, supported by RT-qPCR. What’s more, we predicted that these two mRNAs were targeted by miR-141-3p and miR-200a-3p from 3 databases and were negatively correlated in BC. Thus, we predicted that lncRNA OCIAD1-AS1 might interact with miR-141-3p/miR-200a-3p to regulate GPM6B/AKAP11 to participate in mechanisms in bladder cancer. A large number of subsequent experiments should be conducted to confirm this conclusion.

Unfortunately, there are still many shortcomings in our study. We preliminary predicted that two networks of lncRNA OCIAD1-AS1/miR-141-3p/GPM6B and lncRNA OCIAD1-AS1/miR-200a-3p/AKAP 11 may play potential roles in BC. However, more trials are still needed for verfication.

## Conclusion

In general, we identified 6 pyroptosis-correlated lncRNAs OCIAD1-AS1, MAFG-DT, SLC25A25-AS1, SNHG18, PSMB8-AS1 and TRIM31-AS1.And a predictive model was established for BC patients. At the same time, the correlation between pyroptosis-correlated lncRNAs and immune infiltration was elucidated. As for sensitivity of anti-tumor drugs, the high-risk group was more sensitive to Sorafenib, Bicalutamide and Cisplatin, while the low-risk group was more sensitive to AKT.inhibitor.VIII, Salubrinal and Lenalidomide, etc. Our study predicted the network of lncRNA OCIAD1-AS1/miR-141-3p/GPM6B and lncRNA OCIAD1-AS1/miR-200a-3p/AKAP11, which may play a potential role in BC. However, more trials are still needed for verfication, which is also the focus of our future study.

## Data Availability

The original contributions presented in the study are included in the article/Supplementary material, further inquiries can be directed to the corresponding author.
